# Health risk assessment of groundwater use for drinking in West Nile Delta, Egypt

**DOI:** 10.1038/s41598-025-90477-3

**Published:** 2025-03-03

**Authors:** Zenhom E. Salem, Samia S. Hasan, Ahmed M. Sefelnasr

**Affiliations:** 1 Geology Department, Faculty of Science, Tanta University, Tanta, 31527 Egypt; 2 Desert Research Center, El Matariya, 11753 Egypt; 3 National Water and Energy Center, United Arab Emirates University, Al Ain, 15551 United Arab Emirates

**Keywords:** Nile Delta Groundwater, Health risk assessment, Synthetic pollution index, Non-carcinogenic risk, GIS, Statistical analysis, Environmental sciences, Hydrology, Natural hazards

## Abstract

Human health is at risk from drinking water contamination, which causes a number of health problems in many parts of the world. The geochemistry of groundwater, its quality, the origins of groundwater pollution, and the associated health risks have all been the subject of substantial research in recent decades. In this study, groundwater in the west Rosetta Nile branch of the Nile Delta Aquifer is examined for drinking potential. Numerous water quality indices were applied, such as water quality index (WQI), synthetic pollution index (SPI) models, and health risk assessment (HRA) method. The limits of the measured parameters are used to test its drinking validity on the basis of WHO recommendations. TDS in the southern regions is within the desirable to allowable limits with percent 25.3% and 29.33%, respectively. Nearly all the study area has desirable value for HCO_3_, Al and Ba. Ca and Mg have desirable values in the center and south portion of the investigated area, whereas in the north are unsuitable. Na, Cl and SO_4_ fall within the desired level in the regions of the south but become unsuitable towards the north. Mn and NO_3_ are inappropriate except in the northwestern part. Fe is within suitable range in the southwestern and northwestern regions. Pb, Zn, Cu, and Cd were undetected in the collected samples. Regarding to WQI the study area is classified into 4 classes good, poor, very poor and unfit for drinking water from south to north. According to SPI model, 20%, 18.7%, 18.7%, 8% and 34.6% of water samples are suitable, slightly, moderately, highly polluted and unfit, respectively from south to north. Based on HRA, Children are the most category endangered with percent 14.7% of the overall samples obtained, followed by females and males with percent 12% and 8%, respectively. This study offers insights into the conservation and management of coastal aquifers’ groundwater supplies. These findings have significant implications for developing strategies and executing preventative actions to reduce water resource vulnerability and related health hazards in West Nile Delta, Egypt.

## Introduction

In recent years, rapid urbanization and population growth, stress on natural resources, and global climate change have caused the demand for water to increase. Sustainable water resource management is becoming increasingly important to meet this demand. It is critical to manage water resources globally since groundwater is essential for meeting human needs and for sustaining life^1–4^. Furthermore, unregulated exploitation of groundwater resources has resulted in water shortages over recent decades, which has adversely affected groundwater quality and levels^5–10^. Salinization is a significant issue in many coastal regions globally, particularly in semi-arid and arid areas. It is seen as a crucial and visible issue that threatens future water resources and reduces water quality. Groundwater salinization is a key concern because it restricts water availability for both agricultural and urban needs, impacting the resilience and sustainability of coastal areas. An increase in total dissolved solids (TDS) or chloride (Cl) levels is a clear indicator of salinization^11–13^. The issue of water quality has garnered significant attention in coastal aquifers worldwide due to the aforementioned reasons for example, Thriassion Plain and Eleusis Gulf, Greece^14^, north Kuwait^15^, China^15^, Bangladesh^16^, Spain^17^, Mexico^18^, and others. As groundwater quality is equally important as its quantity, it is crucial to carefully assess it.

Heavy metals pose a toxic threat to human health and ecosystems when their concentrations surpass established limits as they can disrupt ecological systems, endanger human health, and worsen the quality of groundwater. Specific heavy metals, including copper (Cu), zinc (Zn), manganese (Mn), and chromium (Cr), are vital for metabolic processes in traces quantity, but become hazardous at high levels. In contrast, metals such as cadmium (Cd) are toxic even at minimal concentrations^19,20^. Tackling the presence of trace element-contaminated water resources is crucial for protecting both the ecosystem and human health^21^. Additionally, understanding the environmental behavior of these trace elements, including their transfer, fate, persistence, and the health risks they pose to consumers through the food chain, is vital. The health impacts of these elements are significantly influenced by factors such as their behavior, specific chemical composition, and binding state. Gaining insight into these factors is the key to assessing the potential risks of trace elements and devising effective strategies to minimize their harmful effects^22,23^. Controlling and mitigating these harmful effects can be achieved by monitoring heavy metal distribution, concentration, and health risks regularly.

Evaluating groundwater quality is a fundamental approach for ensuring the sustainable management of this essential resource. Various methodologies have been employed to assess groundwater quality, including stoichiometric, graphical, index-based, and inferential chemometric techniques, which are commonly used to analyze and monitor groundwater conditions and hydrogeochemical properties^24–26^. Additionally, advanced tools such as clustering, regression analysis, neural networks, and machine learning algorithms have been incorporated to observe and predict water quality trends effectively^27–29^. Given the variety of hydrochemical criteria, the water quality index (WQI) technique serves as an effective tool for evaluating groundwater quality. Due to its comprehensive calculation method, assessing groundwater quality through multiple hydrochemical parameters is considered a more reliable and robust approach. As a result, WQIs have been widely utilized in groundwater quality assessments. The most frequent techniques for assessing water quality are the WQI for drinking and synthetic pollution index (SPI). The Water Quality Index (WQI) for drinking water and the Synthetic Pollution Index (SPI) are effective tools for measuring and evaluating overall water quality, offering a more comprehensive approach than traditional techniques for evaluating the quality of water. Each of the two types of standard water quality index models (WQI and SPI) measure the cumulative impact of different physicochemical variables on groundwater quality based on weight and rate. Each physicochemical parameter is weighed according to its influence on drinking water quality^30,31^. Since many people rely on groundwater for drinking and other household purposes, high levels of nitrate in drinking water can result in serious health risks^32–34^. Therefore, health risk assessment (HRA) based on nitrate concentration was applied as drinking water quality criteria^35–37^. Combining water quality indices with GIS techniques provides the most effective method for detecting and visualizing changes in groundwater facies. Several studies have applied water quality indices (WQI and SPI) and HRA methods to evaluate groundwater quality for human use in various regions, and these techniques have proven successful. For instance, studies have been conducted on Makkah Al-Mukarramah Province (Saudi Arabia)^38^, coastal plain in Nigeria^23^, dumpsite in Awka (Nigeria)^22^, El Fayoum depression (Egypt)^30^, El Kharga Oasis (Egypt)^39^, and Central Nile Delta Region (Egypt**)**^40^.

The quaternary aquifer, coastal aquifer, is considered the main source of groundwater in the area west of Rosetta branch. Based on the previous studies, the groundwater within the study area exposed to several factors, which may lead to increase signs of groundwater quality deterioration. These factors are mainly attributed to anthropogenic activities and sea water intrusion^12^. Moreover, most previous studies conducted west of the Nile Delta have primarily focused on the morphological and geological features of the terrain^41–44^. Additionally, water sources have been examined in terms of their geochemical properties and suitability for irrigation purposes^8,12,45,46^. However, limited attention has been given to evaluating the quality of groundwater for drinking purposes within the study area. As a result, significant knowledge gaps remain regarding the suitability of groundwater for human consumption in this region.

Based on the aforementioned objectives, this study aimed to evaluate the quality of groundwater for drinking purposes in the region west of the Nile Delta’s Rosetta branch. This study was conducted to develop geospatial maps of physicochemical parameters in groundwater to determine the quality suitability of drinking water. Furthermore, in order to assess the water quality from the aspect of human health, two typical water quality index models are used, namely water quality index (WQI) and synthetic pollution index (SPI). In order to analyze the data concerning water quality, descriptive statistics and correlation matrices were applied. Eventually, human health risk (HRA) was assessed in the study region via contaminated water consumption by adults (males and females) and children. It is expected that this study will assist decision makers in identifying vulnerable zones and optimizing monitoring networks for groundwater quality.

## Materials and methods

### Study area

**Fig. 1:**
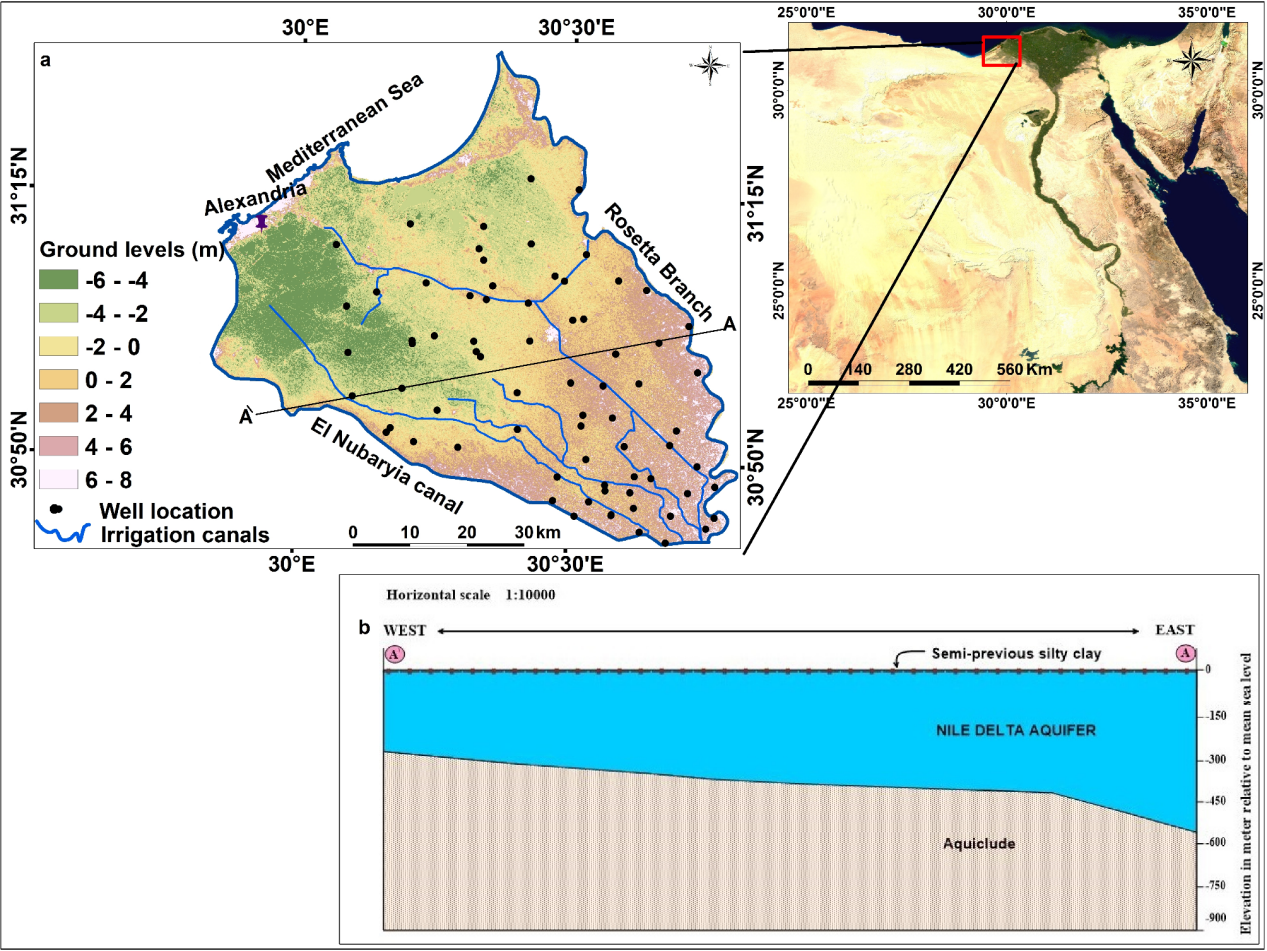
(a) Location of the study area, (b) Hydrogeological cross section of the research region^53^.

The research area is situated in the northwest section of the Rosetta Nile branch. Figure 1 displays the general location map of the research region, which is bounded to the west with Rosetta branch, to the east with El Nubaryia canal, and to the north with the Mediterranean Sea coast. Several authors have discussed the geomorphology, geology and hydrogeology of the western Nile Delta area such as^43,44,47–50^. Geomorphologically, the study area is dominated by young alluvial plain. It has an almost elevated ground surface varying from 8 m above sea level to 6 m below mean sea level (Fig. 1a). The surface is occasionally occupied by aeolian sediments of sand dunes. There are brackish lakes in many locations and water-logged areas in the northern part. On a geological basis, Quaternary sediments are found in the study area, primarily in the Bilqas Formation on the top and the Mit Ghamr Formation below. The composition of the Bilqas consists predominantly of very coarse sand interbedded with molluscan fragments rich in clay^41^. The composition of Mit Ghamr Formation comprises sand and gravels intercalated with clay. The sand is quartz ranging in size from medium to coarse grains, whereas the gravels are composed of flint and dolomites. The formation deposits in general are continental deltaic. On the other side; towards the north marine elements become noticeable^42^. In the Nile Delta, this formation is the main aquifer belonging to the Quaternary–Late Tertiary period. The aquifer structure is capped by a semi-confining layer known as a clay layer and underlined by aquiclude (Fig. 1b).

Near the Rosetta branch, the aquifer’s thickness peaks at around 850 m and then sharply declines in the west, where it significantly reduces close to the El Nubaryia canal. The thickness of the aquifer near the Rosetta branch is at its maximum, around 850 m, and it declines sharply to the west, where it notably diminishes near the El Nubaryia canal^46^. The Quaternary aquifer is replenished through various sources: through river branches, by infiltration from surface water, particularly from the channel systems, and drainage networks. Also, rainfall is another recharge source of the Nile Delta aquifer. The annual rainfall varies from 50 mm/yr in the south to around 200 mm/yr in the north^51^. Natural discharge of groundwater occurs through drainage, evapotranspiration, or artificial discharge occurs via direct well extraction, which is the main discharge method for the aquifer. The average annual evapotranspiration in the study region is 1680 mm^52^.

### Groundwater sampling and physicochemical parameters

In the research region, seventy-five groundwater samples were obtained at various locations. Samples were gathered from the region’s existing hand pumps and boreholes (Fig. 1). During the sampling process, all spots were georeferenced. Using standard procedures, all water samples were examined for numerous physicochemical features, comprising total dissolved solids (TDS), sodium (Na), potassium (K), calcium (Ca), magnesium (Mg), sulfate (SO_4_), bicarbonate (HCO_3_), chloride (Cl), nitrate (NO_3_), aluminum (Al), iron (Fe), barium (Ba), manganese (Mn), lead (Pb), zinc (Zn), copper (Cu), cadmium (Cd), pH, and total hardness (TH). The physicochemical analyses were conducted at the Geology Department Laboratory, Faculty of Science, Tanta University, as well as at the Center for Scientific Research and Measurements, Tanta University. Groundwater samples were taken in one-litre polythene bottles using conventional sampling procedures. Deionized water was used to properly clean and rinse the bottles in order to eliminate any possible contaminant^54^. pH and TDS meters were used to measure pH and TDS, respectively, in situ^55,56^. However, TH, Cl, and HCO_3_ were obtained in the lab using the titration procedure^57^, whereas Ca, Mg, Na, K, Al, Fe, Ba, Mn, Pb, Zn, Cu, and Cd were determined via Inductive Coupled Plasma (ICP)^58^. No traces of Pb, Zn, Cu, or Cd were found in the samples collected. A spectrophotometer was used to quantify nitrate and sulphate (Hach’s Direct Reading)^57^. Table 1 illustrates the summary of statistical data of the various physicochemical variables’ detected concentrations. In order to validate analytical methods, the devices were calibrated and accuracy of the tested samples was assessed. The accuracy of the ion concentration measurements in meq/L was verified by computing the charge error balance (CBE)^59,60^. All examined samples had a CBE within the allowable range of ± 5%.

### Evaluation of water quality on the basis of water quality index models

To assess the overall quality of water for drinking, a water quality index model becomes a necessity and accepted tool. In the current investigation; the water quality index (WQI), the synthetic pollution index (SPI) models and health risk assessment (HRA) were used to properly assess the quality of water for drinking. The spatial distribution of the physicochemical parameters according to^61,62^ desirable and permissible limits and water quality index maps were interpolated using Kriging technique in ARC GIS 10.8 software.

#### Water quality index model (WQI)

The specifications of water for drinking according to^61,62^ based on chemical analysis including the acceptable ranges of the variables quantified are provided in Table 1. The steps and the equations of WQI calculations are described as follow (Table 2):

Step 1 is to calculate Constant of proportionality (ki) .………............Equation 1

Step 2 includes calculation of Weight coefficient (wi) ……................Equation 2

Step 3 involves Relative weight calculation’s (Wi)…………...............Equation 3

Step 4 includes Water quality rating calculation’s (qi) .……...............Equation 4

Step 5 is to established drinking Water quality index (WQI) ……….….Equation 5

**Table 1:** Statistical evaluation of the collected hydrochemical data and water suitability for drinking^61,62^.

Classification Parameter	Statistical analysis		Classification according to WHO guidelines		No. of samples	% of samples
	mg/L					
pH	Min	5.05	Desirable limit	6.5–8.5	52	69.33%
	Max	8.7	Allowable limit	8.5		
	Av	7.16				
TDS	Min.	190	Desirable limit	500 (mg/L)	19	25.3%
	Max.	27,680	Allowable limit	1000 (mg/L)	22	29.33%
	Av.	3806.2				
TH	Min.	25.93	Desirable limit	300 (mg/L)	23	30.66%
	Max.	3315	Allowable limit	600 (mg/L)	23	30.66%
	Av.	762.81				
Na	Min.	37.98	Desirable limit	200 (mg/L)	35	46.66%
	Max.	9200	Allowable limit	200 (mg/L)		
	Av.	1156.7				
K	Min.	2.34	Desirable limit	12 (mg/L)	50	66.66%
	Max.	337.7	Allowable limit	12 (mg/L)		
	Av.	34.86				
Ca	Min.	4.74	Desirable limit	200 (mg/L)	61	81.33%
	Max.	670	Allowable limit	200 (mg/L)		
	Av.	153.62				
Mg	Min.	3.43	Desirable limit	125 (mg/L)	58	77.33%
	Max.	430	Allowable limit	125 (mg/L)		
	Av.	92.38				
HCO_3_	Min.	13.02	Desirable limit	350 (mg/L)	71	94.66%
	Max.	394	Allowable limit	350 (mg/L)		
	Av.	222.68				
SO_4_	Min.	0	Desirable limit	250 (mg/L)	49	65.33%
	Max.	2300	Allowable limit	250 (mg/L)		
	Av.	365.7				
Cl	Min.	48	Desirable limit	250 (mg/L)	35	46.66%
	Max.	14,300	Allowable limit	250 (mg/L)		
	Av.	1844.1				
NO_3_	Min.	0	Desirable limit	10 (mg/L)	42	56%
	Max.	202	Allowable limit	50 (mg/L)	22	29.33%
	Av.	22.92				
Al	Min.	0.0009	Desirable limit	0.2 (mg/L)	72	96%
	Max.	0.356	Allowable limit	0.2 (mg/L)		
	Av.	0.069				
Fe	Min.	0.0001	Desirable limit	0.3 (mg/L)	46	61.33%
	Max.	34.56	Allowable limit	1 (mg/L)	13	17.33%
	Av.	1.03				
Ba	Min.	0.0001	Desirable limit	1.3 (mg/L)	74	98.66%
	Max.	15.39	Allowable limit	1.3 (mg/L)		
	Av.	0.32				
Mn	Min.	0.0009	Desirable limit	0.1(mg/L)	18	24%
	Max.	2.85	Allowable limit	0.4 (mg/L)	18	24%
	Av.	0.56				

**Table 2:** Equations used in calculation of WQI, SPI and HRA.

Equation	No.	Definitions
$\:{k}_{i}=1/\sum\:_{i=0}^{n}1/{s}_{i}$ ^31^	1	Ki Constant of proportionality
$$\:{w}_{i}=\raisebox{1ex}{${k}_{i}$}\!\left/\:\!\raisebox{-1ex}{${s}_{i}$}\right.$$ ^63^	2	Si, measured in mg/L, is the WHO drinking water criterion. n is the total number of the analyzed samples
$\:{W}_{i}={w}_{i}/\sum\:_{i=0}^{n}{w}_{i}$ ^63^	3	wi weight coefficient of each parameter
$\:{q}_{i}=\frac{{v}_{i}}{{s}_{i}}\times\:100$ ^31^	4	Wi relative weight of the parameter
$\:WQI=\sum\:{q}_{i\:}\times\:{W}_{i}$ ^8^	5	qi water quality rating of the parameter
$\:SPI=\sum\:_{i=1}^{n}\frac{{v}_{i}}{{S}_{i}}\times\:{w}_{i}$ ^54^	6	vi is the concentration of parameters (mg/L) WQI water quality index SPI synthetic pollution index
$\:{Intake}_{oral}=\frac{{C}_{w}+IR+EF+ED}{BW+AT}$ ^64^	7	Intake_oral_ the mean daily exposure dosage from groundwater consumption (mg/kg/day) Cw the concentration of a particular pollutant in groundwater IR groundwater ingestion rate
$\:{HQ}_{oral}=\frac{{Intake}_{oral}}{{RFD}_{oral}}$ ^65^	8	EF the exposure frequency ED the exposure duration BW the average body weight AT average exposure time HQoral hazard quotient RfDoral reference dosage for noncarcinogenic contaminant through drinking water intake, RFD of NO_3_ = 1.6 mg/kg/day ^50^

#### Synthetic pollution index model (SPI)

The calculations of SPI involve the steps outlined below^66,67^, Table 2 includes the calculation equations:

Step 1: Constant of proportionality (Ki)………………..... Equation 1

Step 2: Weight coefficient (wi)……………………….........Equation 2

Step 3: Synthetic pollution index (SPI) ………………......Equation 6

In accordance with SPI, the water quality is divided into five distinct categories. The SPI classifications include unfit for human consumption, highly polluted, moderately polluted, slightly polluted, and suitable as shown in Table 3. In the present work the findings of the chemical and physical analysis of groundwater samples such as EC, pH, TDS, Ca, Mg, Na, K, HCO_3_, TH, Cl, SO_4_, NO_3_, Al, Fe, Ba, and Mn are utilized for the calculations of both WQI and SPI models.

**Table 3:** Classification of samples based on SPI^66,68^.

Type of water	Range
suitable	< 0.2
slightly polluted	0.2–0.5
moderately polluted	0.5–1
highly polluted	1–3
unsuitable for drinking purposes	> 3

#### Health risk assessment (HRA)

An approach for determining the risk to human health has been developed by the US Environmental Protection Agency^69^ which has been vastly applied^64,70,71^. Significantly over the past decade, the potential dangers to human health have grown, because of the ingestion of contaminated water particularly in regions where groundwater is considered a main source for drinking. It is therefore necessary to review the HRA, which is a crucial reference in preserving and managing the groundwater environment^70,72^.

Water affects the human health, which is the main source of the threat to public health. Hence, as a key target, this study is aimed at drinking water intake; particularly NO_3_ has been applied as the HRA factor and noncarcinogenic hazard is computed through Eq. 7 and Table 4. The hazard quotient (HQ) is applied using Eq. 8 to assess nitrate risk, Table 2.

**Table 4:** Standard limits of HRA variables^69,73^.

	Gender and age group		
	Male	Female	Children
IR (L/day)	1.5	1.5	0.7
EF (days/year)	365	365	365
ED (years)	30	30	12
BW (kg)	65	55	18.5
AT (days)	10,950	10,950	4380

If HQ oral is greater than 1, the groundwater is considered a source of noncarcinogenic hazard, whereas HQoral’s safe value is less than 1^69,74^.

## Results and discussion

### Physicochemical parameter-based analysis

Drinking water is soft, low in TDS, and free of dissolved toxic components. The groundwater quality assessment is based on^61^ drinking water standards. Table 1 shows the statistical analysis of the evaluated physicochemical parameters that were utilized to define the analyzed groundwater drinking quality. Water’s pH value indicates how acidic or alkaline it is. If the pH reading in drinking water exceeds the recommended range, it can lead to symptoms such as nausea and vomiting^75^. The examined groundwater’s pH value varies from 5.05 to 8.7, with an average of 7.16. The desired limits are met by 52 of the groundwater samples with percent 69.33% of total collected samples. The pH values reported by Sharaky et al.^49^ for groundwater in coastal areas of Egypt’s western Nile Delta align with the results of this investigation.

**Fig. 2:**
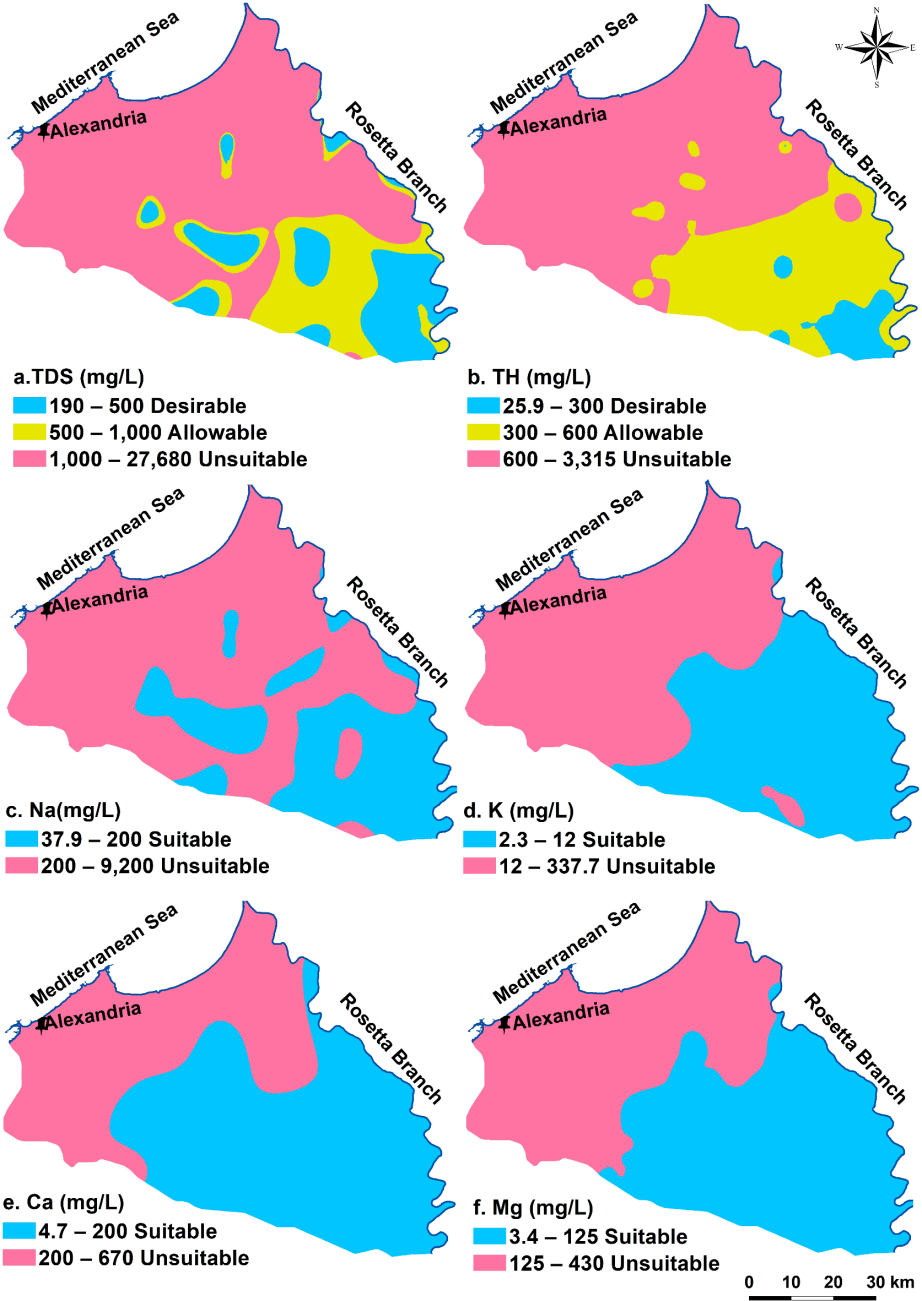
Groundwater drinking suitability spatial patterns depend upon concentrations of (a) TDS, (b) TH, (c) Na, (d) K, (e) Ca, and (f) Mg.

TDS concentration is a crucial factor in evaluating water quality. It results from the presence of dissolved organic and inorganic substances in the water^39^. The average TDS value is 3806.28 mg/L, with measurements varying from 190 to 27680 mg/L. According to WHO 2011, 45.4% of the 34 wells are inappropriate water for drinking, 29.3% of the wells (22 wells) are within the allowed limit, and 25.3% of the wells (19 wells) have desired concentrations. Samples with unsuitable TDS values are plotted in the north of the investigated region, while samples with desirable and allowable characters are found in the southern part (Fig. 2a). The higher TDS concentrations in groundwater are largely attributed to seawater intrusion, which becomes more pronounced moving northward. However, in the south and center parts of the research region, fresh to slightly saline water helps dilute the groundwater, and lower TDS values are found close to the Rosetta branch and nearby irrigation channels^12^. A study by^76,77^ also found higher TDS in groundwater in the eastern Nile Delta, Egypt. TH values vary from 25.93 to 3315 mg/L with a mean of 762.81 mg/L. Twenty-three wells (30.6%) of the groundwater wells fall in the desirable range; 30.6% of the wells (23 well) are within the allowable limit and 29 wells (38.8%) are situated in the north part and occupy most of the research area (Fig. 2b). Mansour et al., 1983 observed a similar pattern of hardness in collected groundwater of the east Nile Delta, Egypt.

In the research area’s groundwater, sodium is the most common positive ion, followed by calcium, magnesium, and potassium. The significant amounts of sodium and calcium are ascribed to ion exchange processes among minerals. In clastic sediment aquifer, groundwater derives its calcium from the dissolution of silicate minerals like anorthite^12,76^. Na contents show variation in the amount from 37.98 to 9200 mg/L, averaging 3806.28 mg/L. Thirty-five wells (46.66%) of the collected samples fall with the desirable level, these samples are allocated in the southern parts. While forty wells (53.34%) exceed the desirable limit (Table 1; Fig. 2c). The values of K vary from 2.34 to 337.7 mg/L averaging 34.86 mg/L. Fifty wells fall with the desirable level and represent 66.66% of total wells, on the other hand twenty-five wells show K concentration over the dispersible limit (Table 1). K contents in 66.66% of the obtained samples from the southern regions fall within the desired range (Fig. 2d). With an average value of 153.62 mg/L, the detected Ca in samples varies from 4.74 to 670 mg/L. The Ca content of sixty-one (81.33%) of the groundwater samples that were gathered is within the desired range. In the northern parts (18.67%) have Ca content over the suitable limit (Table 1; Fig. 2e). The average value of the magnesium ions is 92.38 mg/L, with a range of 3.43 to 430 mg/L. Fifty-eight wells (77.3%) are below the desirable limit and found in the south region. The northern parts are occupied by seventeen wells which have high Mg content exceeds the suitable limit (Table 1; Fig. 2f).

**Fig. 3:**
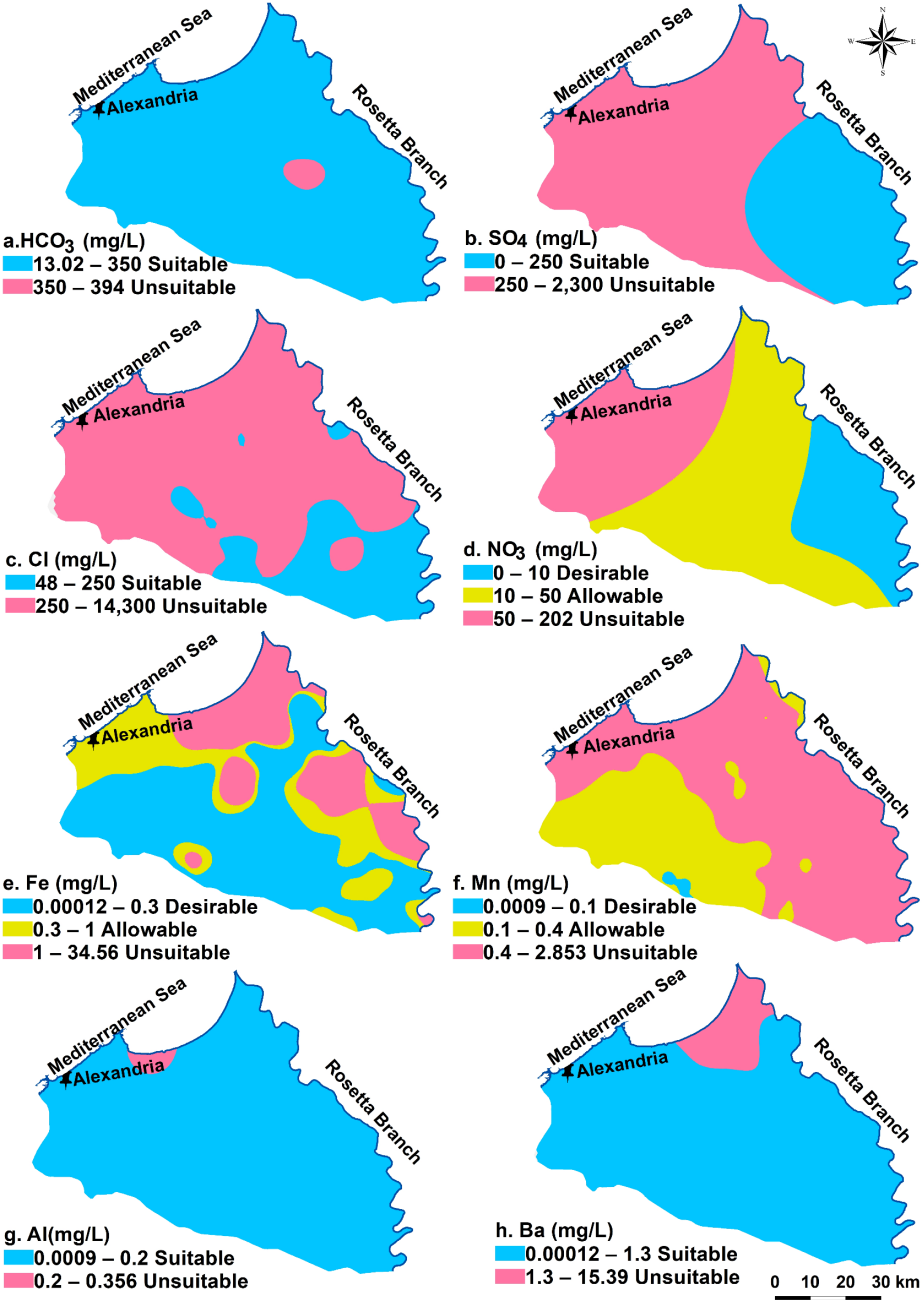
Spatial allocation of the gathered sample quality dependent on (a) HCO3, (b) SO4, and (c) Cl, (d) NO3 content, (e) Fe, (f) Mn, (g) Al and (h) Ba content.

It was found that the amount of HCO_3_ in the obtained samples ranges from 13.02 to 394 mg/L with an average value of 222.68 mg/L. Most of the study area (71 wells, 94.66%) exist within the desired values, and 4 wells find over the limit (Table 1; Fig. 3a). The analysis of SO_4_ in the groundwater samples show that the ion concentrations range from 0 to2300 mg/L with a mean of 365.7 mg/L. 49 wells (65.33%) are found within the desired level of 250 mg/L and twenty-six wells are located to the northern parts are above the limit (Table 1; Fig. 3b). The average chloride concentration is 1844.09 mg/L, with a range of 48 to 14300 mg/L. Of the wells, 35 (46.66%) fall inside the desired range, and forty wells (53.34%) exceed the limit and found at the north part (Table 1and Fig. 3c). A range of 0 to 202 mg/L of NO_3_ has been reported, with the average being 22.92 mg/L. Forty-two and twenty-two samples have NO_3_ content below the desired and allowable range and represent 56% and 29.33% of the total samples, respectively (Table 1). 14.67% of groundwater samples of high NO_3_ content are recognized in the coastal part of the region (Fig. 3d). Since the aquifer under investigation consists of clastic sediments, the synthesis of bicarbonate ions results from the generation of CO_2_ due to the disintegration of organic material in the southern region, along with the deposition of carbonate minerals in the northern areas. Additionally, the increase in sulfate and chloride minerals in the northern parts is attributed to seawater intrusion^78,79^. The higher nitrate concentrations are primarily linked to agricultural activities, particularly the overusing of fertilizers and the reutilize of wastewater for watering the agricultural lands^7,45^.

The average content of Fe is 1.03 mg/L, with values detected from 0.00012 to 34.56 mg/L. Forty-six samples (61.33%) have the desired range (0.3 mg/L), 13 wells are within the allowable range (17.33%) and 14 wells (21.34%) are unsuitable (Table 1; Fig. 3e). Samples of unsuitable Fe content are mainly found in the eastern regions and some parts in the northeastern areas. The results indicated that Mn cations vary from 0.0009 to 2.85 mg/L with an average concentration of 0.56 mg/L. Just 18% of total samples have desired concentrations, 18% fall within the allowable level, and 64% are outside the limit. The groundwater that is appropriate has been distributed in the northwestern region (Table 1; Fig. 3f). The concentrations of iron and manganese that exceed acceptable levels may be due to the fertilizer application and the sewage drainage^12,45^. Except three wells, Al concentrations of almost gathered samples have desired values, having an average concentration of 0.069 mg/L and a range of 0.0009 to 0.356 mg/L. (Table 1; Fig. 3g). The range of the Ba content is 0.0009 to 2.85 mg/L, with an average concentration of 0.56 mg/L. The majority of the wells (74 wells, or 98.66%) fall within the range that is allowed (0.5 mg/L, Table 1; Fig. 3h).

### WQI model

**Fig. 4:**
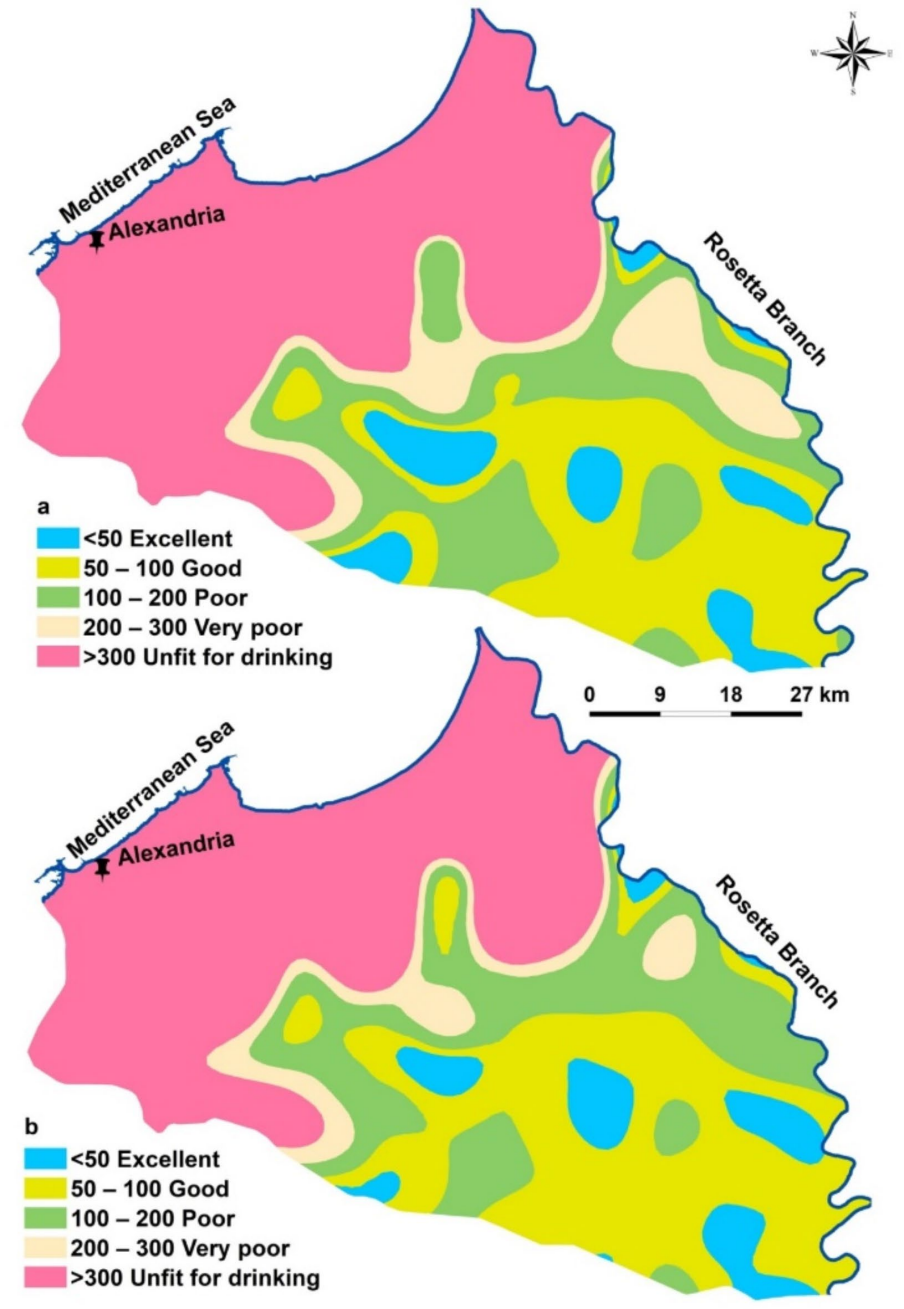
Groundwater suitability spatial distribution maps for drinking based WQI. (a) based on desirable limits and (b) based on allowable Limits.

In accordance with Eq. (5), WQI models were utilized to evaluate groundwater quality across the investigated area, classifying it based on commonly reported water quality indices. The Drinking Water Quality Index (WQI) was implemented to find out whether groundwater in the research territory was suitable for human consumption. According to^61^, water quality index of the obtained samples in the research region is categorized into five classes (Table 5; Fig. 4). Based on the desirable limit, the south regions of the concerned area have excellent (7 wells, 9.3%) to good water quality (29wellls, 38.6%). The poor water quality class represents 21.3% and occupies almost of the central parts. The samples from the northern part have very poor to unfit characters represent 10.6% and 20.2% of the total samples, respectively (Table 5; Fig. 4a). Depending on the allowable range as shown in Fig. 4b, the south and center parts of the investigated area have excellent (10 wells, 13.4%) to good water quality (30 wells, 40%). In the northern part of the study area, groundwater quality is classified as 20% poor, 8% very poor, and 8% unfit for drinking (Table 5). This is due to elevated sodium (Na) and chloride (Cl) concentrations caused by invasion of Mediterranean seawater, evaporation, and ion exchange processes.

**Table 5:** Samples classification according to^61^.

		Desirable limit		Allowable limit	
Type of water	Range	No. of wells	% of wells	No. of wells	% of wells
Excellent water	< 50	7	9.3	10	13.4
Good water	50–100	29	38.6	30	40
Poor water	100–200	16	21.3	15	20
Very poor water	200–300	8	10.6	6	8
Unfit for drinking water	> 300	15	20.2	14	18.6

The matrix of correlation describes the relationship between WQI and various variables of water quality (Table 6). The higher correlation coefficient, the greater negative impact of elements will be on drinking water quality. TDS, EC, TH, Ca, Na, Mg, K, Cl, and SO4 are found to have a strong positive correlation with the reduction in water quality (greater WQI). By rising TDS, which is the mean factor for quality degradation, It indicates that each of these factors rises. NO_3_ and Fe show moderate positive relationship with WQI, while pH has a moderate negative effect on the groundwater quality. HCO_3_ has very weak negative correlation values with WQI. There is a positive weak relationship between Al and Mn with groundwater quality.

**Table 6:** Matrix of correlations between the regulatory factors and WQI.

Parameter	WQI
TDS	**0.99**
pH	*-0.38*
TH	**0.96**
Ca	**0.93**
Mg	**0.95**
Na	**0.99**
K	**0.84**
HCO_3_	***-0.09***
SO_4_	**0.78**
Cl	**0.99**
NO_3_	*0.38*
Al	***0.01***
Fe	*0.48*
Mn	***0.04***

Values listed in red font are strong correlation, blue font are intermediate correlation, values listed in green font are weak correlation.

### SPI model

**Fig. 5:**
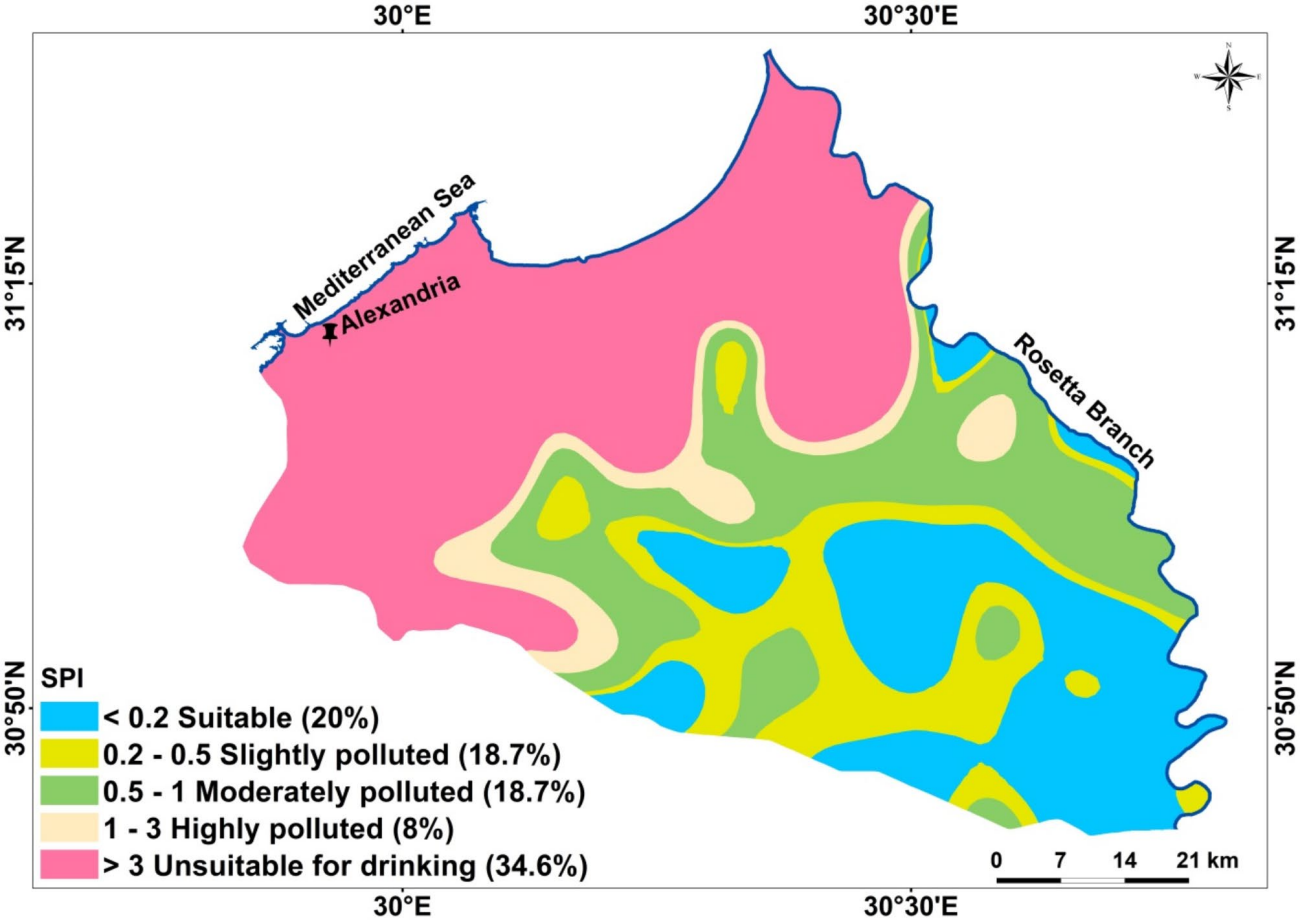
Spatial distribution groundwater quality maps according to the SPI model.

Figure 5 shows the results are achieved by analyzing water samples for groundwater quality evaluation and categorization using SPI and its spatial distribution. Depending on the SPI model the obtained samples were classified, nearly 20% as suitable, 18.7% as slightly polluted, 18.7% as moderately polluted, 8% as highly polluted and 34.6% as unsuitable for human consumption (Fig. 5). According to the interpolated GIS map of SPI, it is clear that the southern part is suitable to slightly polluted. While towards the north the groundwater graded from moderately to highly polluted until become unsuitable near the coastal zone (Fig. 5). One of the potential reasons of such pollution is the invasion of the Mediterranean Sea water into the studied aquifer.

**Fig. 6:**
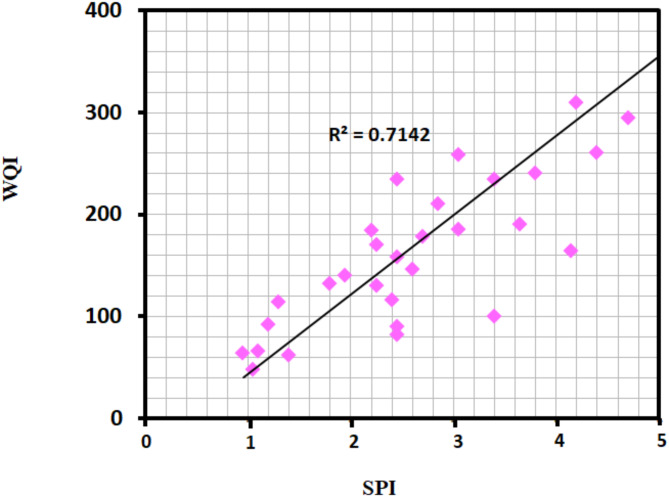
Correlation between WQI and SPI.

To indicate the relationship between the WQI and SPI indices, ArcGIS10.8 was used to convert the maps of both these indices into raster with a same pixel size and resolution of 250 m×250 m. Twenty-nine points are distributed in the study area and covered all the classes for WQI and SPI raster. The results of these points are used to establish the relationship between two indices. Figure 6 shows a strong relationship between the two indices (r^2^ = 0.7142). A comparison of the WQI and SPI models reveals that the quality of drinking water for drinking is declining in areas close to the Mediterranean Sea. Furthermore, the suitable zones in the southern region of the study area appear in the same locations on both index maps (Figs. 4 and 5).

### Health risk assessment (HRA)

In many countries where groundwater is the main supply of drinking water, there is concern about noncarcinogenic potential of NO_3_^-^^35,65,80^. NO_3_^-^ contaminated groundwater is recognized as a noncarcinogenic hazard to the health of children, males, and females. HRA is expressed as hazard quotient (HQ) and is computed by Eq. 8. If HQ value is higher than one, groundwater become noncarcinogenic risk while the healthy rang is below one. Adult men’s HQ ranges from 0 to 2.91, adult women’s HQ ranges from 0 to 3.44, and children’s HQ ranges from 0 to 4.78, with an average of 0.33, 0.39, and 0.54 for males, females, and children, respectively (Table 7).

**Table 7:** HQ results for the analyzed samples.

Indices	HQ (males)	HQ (females)	HQ (children)
Min.	0.00	0.00	0.00
Max.	2.91	3.44	4.78
Av.	0.33	0.39	0.548
SD.	0.53	0.63	0.87

**Fig. 7:**
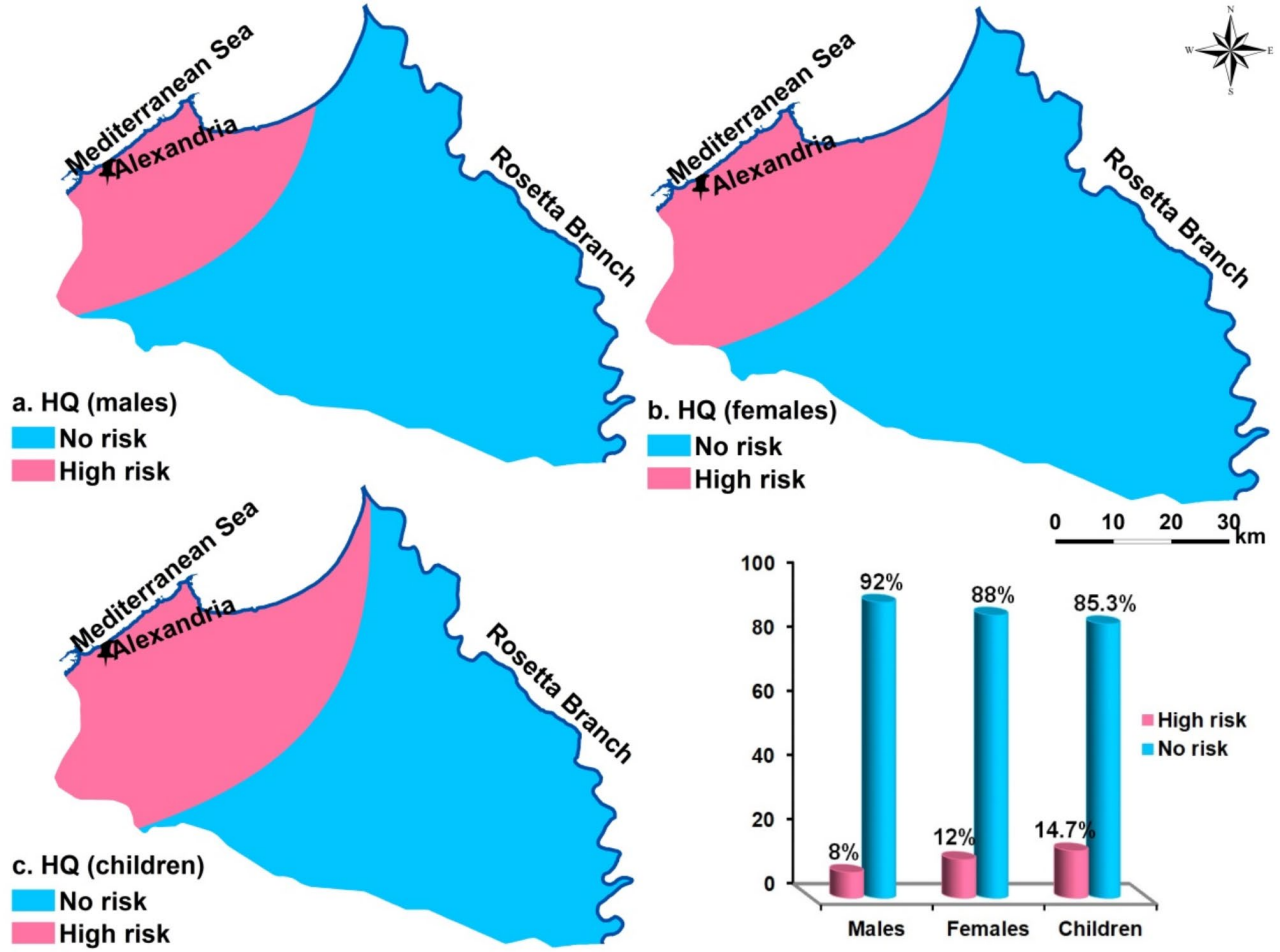
Spatial distribution of Health risk assessment of groundwater samples. (a) males, (b) females and (c) children.

Most of groundwater samples are within the acceptable limit for noncarcinogenic risk (< 1); adult males are the least vulnerable to nitrate contaminations in drinking water (92%), females and children come next (Fig. 7). Adult males and females are harmed at a rate of 8% and 12%, respectively. According to the HQ risk assessment, Children are considered to have the most detrimental effects on human health, followed by females and males (Fig. 7). With the exception of the northern portion, the research region falls under the acceptable non-carcinogenic risk level, according to the spatial distribution of HQ (Fig. 7)^19,39^. reported similar findings in other regions of Egypt, indicating that children are at a higher non-carcinogenic risk than adults. Agricultural activities in the study area expanded from 66 to 80.35% between 1984 and 2011^7^, leading to an increase in nitrate concentrations in groundwater. Drinking water with excessive amounts of nitrate is particularly harmful to children’s health. Previous studies^81,82^ have shown that prolonged exposure to high nitrate concentrations through drinking water can primarily result in methemoglobinemia.

Numerous studies have assessed water quality in similar regions using human health risk models and the water quality index (WQI), and our findings align with these studies. For example, Gad et al.^19^ reported that groundwater in the El-Menoufia region, located in the central Nile Delta, Egypt, contained elevated levels of certain metals, particularly manganese and lead, exceeding the permissible limits for drinking water. Additionally, 42% of the samples posed significant non-carcinogenic health risks through oral exposure, primarily due to high nitrate concentrations. Similarly, Gad et al.^39^ found that the majority of groundwater samples (73.6%) in the El Kharga Oasis, Egypt, were unsuitable for drinking based on the WQI. Health risk models further revealed elevated levels of heavy metals, particularly iron and manganese, with average concentrations exceeding the World Health Organization’s (WHO) recommended limits for drinking water. Moreover, Salem et al.^40^conducted a study using indexical and statistical methods to assess the quality and suitability of water resources for domestic and drinking purposes in the central Nile Delta, Egypt. The findings indicated that 51% of the water in the northern region was unsuitable for these uses. Furthermore, concentrations of manganese, iron, and zinc in the southwestern corner of the area exceeded the desirable limits set by WHO guidelines. Globally, numerous studies have successfully utilized indexical and health risk models to assess groundwater quality, demonstrating their effectiveness in water quality evaluation. For example, Aralu et al.^22^ identified the dumpsite area as the most polluted among the water samples collected from Awka, Nigeria. Their findings revealed that cadmium and lead levels exceeded the threshold limits set by both WHO and Nigerian standards for potable water quality. Additionally, manganese and nickel concentrations were found to surpass the limits specified by Nigerian drinking water quality standards. Ayejoto et al.^26^carried out a study to assess the safety of groundwater usage for domestic and drinking purposes in the Nnewi and Awka regions of Nigeria, focusing on oral and dermal exposure pathways and the associated health risks to humans. Children and the elderly are more susceptible to potential health risks compared to middle-aged individuals due to their higher consumption and absorption rates of metals. For water samples from Nnewi, the order of metal absorption is Cd > Pb > Cu > Fe, while for samples from Awka, it is Pb > Cd > Cu > Fe.

Our study adds to the existing literature by offering more detailed and specific insights into the groundwater quality of the western Nile Delta, Egypt. In addition to supporting the conclusions of previous studies regarding the presence of potentially harmful elements, we identified vulnerable zones in the groundwater, highlighting areas that require monitoring to protect water quality. Our research also pinpointed the specific sources of contamination contributing to high contaminant levels in the groundwater. Through these findings, we aim to provide valuable information to decision makers enabling the implementation of effective remediation strategies to ensure the safety of groundwater for human consumption.

## Limitations of study, recommendations, and perspectives for future research

This study revealed a significant negative impact on the physicochemical properties of groundwater in the northern and central areas of the west Nile Delta, Egypt. These issues are primarily attributed to the intrusion of Mediterranean seawater into the aquifer and the excessive use of pesticides and fertilizers in agriculture, which generate high levels of pollutants and elevate the concentrations of dissolved ions above the WHO-recommended limits. The findings indicate that using untreated groundwater for domestic purposes could pose serious health risks, especially in the southern and central regions of the study area. Therefore, it is essential to evaluate the health risks associated with drinking groundwater in the west Nile Delta region to mitigate the potential dangers of consuming contaminated water.

One of the study’s limitations is the absence of scenarios that track the spread of contaminants to identify pollution sources and predict future contamination under both natural and human-influenced conditions. Moreover, there is no seasonal physical and chemical analysis of dissolved elements and heavy elements in representative aquifer samples to ensure the spatio-temporal suitability of the studied groundwater for drinking. The following recommendations have been proposed to enhance water quality and prevent further degradation, considering the current state of groundwater contamination in the western Nile Delta.

- A solute transport model is essential to prevent further degradation of groundwater quality and to predict the future behavior of the aquifer under various proposed scenarios.
- A sufficient number of observation wells should be established by policymakers and stakeholders to facilitate the seasonal analysis of water samples, ensuring the water’s suitability for human health.
- It is suggested to raise and sustain groundwater levels through regulated pumping to prevent further deterioration of the aquifer. This can be accomplished by artificially recharging selected wells in the central area of the study, using desalinated groundwater, or treated wastewater as recharge sources.
- Advanced soft computing techniques, such as artificial intelligence algorithms, can be applied to predict potential hazardous elements in the study area. This approach would improve the monitoring and evaluation of groundwater resources, ultimately enhancing management efforts and increasing the likelihood of securing potable groundwater for future generations.
- Awareness campaigns should be conducted to inform the public in west Nile Delta region about contaminated groundwater and its potential impact on human health risks.

## Conclusions

Groundwater is an important source for drinking in the area between Rosetta branch and El Nubaryia canal, west Nile Delta, Egypt. Thus, this study evaluated groundwater quality by combining a number of physicochemical factors and WQI and SPI models, supported with statistical analysis, health risk assessment model, and GIS tools in order to ascertain if groundwater was safe for drinking and what health risks it posed. The analysis of the physicochemical data (including TDS, pH, TH, Na, K, Ca, Mg, HCO_3_, SO_4_, Cl, NO_3_, Al, Fe, Ba, Mn, Pb, Zn, Cu, and Cd revealed that the south part of the area under investigation has acceptable content of these parameters. However, in the northern regions, concentrations of TDS, TH, Na, Ca, Mg, K, SO_4_, Cl, NO_3_, Fe, and Mn exceed acceptable limits, mostly as a result of groundwater processes such ion exchange, weathering of calcium-rich minerals, and seawater intrusion. The elevated nitrate levels are mainly associated with agricultural activities, especially seepage from irrigation channels and excessive application of agricultural fertilizers. Excessive concentrations of iron and manganese are likely due to fertilizer use and sewage sludge disposal. The groundwater samples analyzed did not detect any traces of Pb, Zn, Cu, or Cd.

Various Water Quality Indices, including the WQI for drinking water and the SPI, were evaluated for the groundwater samples. According to the WQI and SPI assessments, the quality of groundwater in the south of the investigated area is suitable, with both indices showing similar results for these locations. However, groundwater quality for drinking deteriorates in the north of the research area near the Mediterranean Sea coast. The human health risk assessment indicates that children face a greater health risk than adults due to nitrate content in the drinking water. The noncarcinogenic risk results display that the hazard potential for children (14.7%), males (12%), and females (8%) have surpassed the acceptable threshold (HRA ≤ 1) set by the United States Environmental Protection Agency. The WQI and SPI models, along with the HRA and geospatial tools, prove to be effective methods for providing comprehensive information on groundwater quality. Based on the findings of this article, it is concluded that groundwater in the south of the examined area is safe for human consumption, while the groundwater in the north region is unsuitable for drinking.

Groundwater pollution can arise from various sources, including natural processes, industrial activities, agricultural practices, and inadequate wastewater treatment systems. In the study areas, factors such as seawater intrusion, excessive use of pesticides and fertilizers in agriculture, leakage from irrigation channels, natural process (ion exchange and weathering processes), and population growth have significantly contributed to the contamination of water sources, especially groundwater. The findings of this study emphasize the urgent need for improved groundwater quality monitoring and the implementation of effective remediation strategies to ensure its safety for domestic and drinking purposes.

This study highlights the importance of enhanced groundwater quality monitoring in the study regions. Identifying pollution sources and contaminants is essential for developing effective remediation strategies. Consistent monitoring can track changes in water quality over time, facilitating timely interventions to prevent contamination and minimize exposure to hazardous substances.

In conclusion, this research enhances understanding of water quality in the study regions by identifying vulnerable zones and comparing findings with previous studies. It highlights pollution sources and impacts, stressing the need for ongoing monitoring and management to protect public health and the environment.

## Data Availability

The Datasets used and/or analysed during the current study available from the corresponding author on reasonable request.
